# Impairment of IFN-Gamma Response to Synthetic Peptides of *Mycobacterium tuberculosis* in a 7-Day Whole Blood Assay

**DOI:** 10.1371/journal.pone.0071351

**Published:** 2013-08-08

**Authors:** Hannah Priyadarshini Gideon, Melissa Shea Hamilton, Kathryn Wood, Dominique Pepper, Tolu Oni, Ronnett Seldon, Claire Banwell, Paul R. Langford, Robert J. Wilkinson, Katalin A. Wilkinson

**Affiliations:** 1 Clinical Infectious Disease Research Initiative, Institute of Infectious Diseases and Molecular Medicine, University of Cape Town, Cape Town, South Africa; 2 Division of Medicine, Imperial College London, Norfolk Place, London, United Kingdom; 3 MRC National Institute for Medical Research, London, United Kingdom; Fundació Institut d’Investigació en Ciències de la Salut Germans Trias i Pujol. Universitat Autònoma de Barcelona. CIBERES, Spain

## Abstract

Studies on *Mycobacterium tuberculosis* (MTB) antigens are of interest in order to improve vaccine efficacy and to define biomarkers for diagnosis and treatment monitoring. The methodologies used for these investigations differ greatly between laboratories and discordant results are common. The IFN-gamma response to two well characterized MTB antigens ESAT-6 and CFP-10, in the form of recombinant proteins and synthetic peptides, was evaluated in HIV-1 uninfected persons in both long-term (7 day) and 24 hour, commercially available QuantiFERON TB Gold in Tube (QFT-GIT), whole blood assays. Our findings showed differences in the IFN-gamma response between 24 hour and 7 day cultures, with recombinant proteins inducing a significantly higher response than the peptide pools in 7 day whole blood assays. The activity of peptides and recombinant proteins did not differ in 24 hour whole blood or peripheral blood mononuclear cell (PBMC) based assays, nor in the ELISpot assay. Further analysis by SELDI-TOF mass spectrometry showed that the peptides are degraded over the course of 7 days of incubation in whole blood whilst the recombinant proteins remain intact. This study therefore demonstrates that screening antigenic candidates as synthetic peptides in long-term whole blood assays may underestimate immunogenicity.

## Introduction

Assay of the T cell response to antigens of *Mycobacterium tuberculosis* (MTB) is a research priority and critical in the evaluation of new antigenic candidates to improve immuno-diagnosis and vaccines. The immunogenicity of candidate antigens is important to decide which should be selected for further studies, and the ability to compare results is important. Various laboratories use different techniques and readout methods to compare the performance of new candidates. Moreover, these potential comparisons are confounded by variations in individual laboratory approaches that use different readout methodologies to define the immunogenicity of such candidates [1].

The measurement of MTB antigen specific T cell Interferon (IFN) -gamma production is used widely for determination of immune recognition in antigen discovery. Early IFN-gamma release assays used purified protein derivative of MTB, which has also been used in the Tuberculin Skin Test (TST) for over one hundred years [2], [3]. Therefore these early assays had compromised specificity due to cross reactivity of the immune response to homologous proteins in tuberculous, non-tuberculous and environmental mycobacteria. Newer IFN-gamma release assays use MTB specific ESAT-6 and CFP-10, two co-expressed immuno-dominant antigens encoded by the region of difference (RD)-1 of MTB. These antigens were first evaluated in a 6-day lymphocyte stimulation test (LST) and found to be highly specific to infer tuberculosis infection [4], [5]. IFN-gamma release assays were subsequently developed that differed from the classical LST in terms of cell type to be stimulated (whole blood or peripheral blood mononuclear cells), the formulation of the antigenic candidate (overlapping synthetic peptides or recombinant proteins), duration of incubation (from <24 hours to 7 days), or the methods used to read out the results (proliferation, ELISA, ELISpot, flow cytometry) [1], [6]–[30]. Proliferation and IFN-gamma production using PBMCs or MTB specific cell lines has been shown to correlate irrespective of the antigenic formulation (recombinant or synthetic overlapping peptides) of ESAT-6 and CFP-10 [5]. IFN-gamma results from the 24 hour ELISpot assay (using PBMCs) and a 72 hour diluted whole blood assay (WBA) were also found to correlate, when recombinant ESAT-6 and CFP-10 were used [9]. Discordance between reported results have been attributed mainly to the variation in the duration of incubation and have been suggested to be a result of the measurement of various components of the T cell response where short-term assays detect circulating effector cells while long-term assays detect the secondary effector cells that differentiate from central memory cells during culture [28], [31]–[34].

In the present study, we compared the commercially available 24 hour QuantiFERON TB Gold in Tube (QFT-GIT) with a widely used 7 day WBA [1], [34], as well as the ELISpot assay, using ESAT-6 and CFP-10 to induce an IFN-gamma response. The performance of these two antigens in the form of synthetic overlapping peptide pools and recombinant proteins in 7 day WBAs was also evaluated. We report differences between the efficacy of the peptide pools and recombinant proteins to induce IFN-gamma in the short-term, compared to the long-term assays, using whole blood. Moreover, we used SELDI-TOF mass spectrometry to show that the peptides are degraded over the course of 7 days of incubation in whole blood whilst the recombinant proteins remain intact.

## Materials and Methods

### Ethics Statement

The University of Cape Town Faculty of Health Sciences Human Research Ethics Committee (REC336/2004; REC 296/2007; REC 245/2009) approved this study. All participants provided written informed consent.

### Study Subjects

Twenty-nine healthy laboratory and clinical staff (volunteers) with probable occupational exposure to tuberculosis were recruited. TST or HIV-1 testing was not performed. None of the volunteers had symptoms of active TB. Ten were male, 19 female, with median age of 27 years and 25 were BCG vaccinated.

### QuantiFERON-TB Gold In Tube Assay

Commercially available QuantiFERON-TB Gold In Tube (QFT-GIT) tests were performed as per the manufacturer’s instructions, with the optical density values at 450 nm used to calculate results using QuantiFERON TB Gold analysis software v2.50 (Cellestis, Australia). A positive result is defined by >0.35 IU/ml of IFN-γ measured in the TB antigen minus nil tube, and negative when this was <0.35 IU/ml.

### Seven Day Whole Blood Assay

A diluted whole blood assay of 7-days’ incubation was performed as previously described [13], [16], [34]. Briefly, whole blood was diluted 1∶10 in RPMI 1640 medium containing 1% (w/v) L-Glutamine. A 48 well (flat bottom) plate was set up with the antigens and controls to a final volume of 1 ml. ESAT-6 and CFP-10 were used as stimulants, either as peptide pools (3 µg/ml of each peptide) or recombinant proteins (5 µg/ml). Phytohemagglutinin (PHA, 5 µg/ml, Sigma) was included as a positive control and RPMI 1640 medium with 1% L-Glutamine as a negative control. The antigens were added at 100 µl per well, followed by 900 µl of diluted blood. The plate was sealed with micro-pore tape to avoid evaporation during incubation at 37°C with 5% CO_2_. After 7 days of incubation, the supernatants were harvested and stored at −20°C until assayed for IFN-gamma by ELISA.

### IFN-gamma ELISA

Detection of IFN-gamma by ELISA was performed as previously described [35], [36] using the mouse anti-human IFN-gamma monoclonal capture antibody (BD Pharmingen 551221) and the biotinylated mouse anti-human IFN-gamma detection antibody (BD Pharmingen 554550). This assay has a dynamic range of approximately 10–3000 pg/ml. The lowest sensitivity of the assay was 14 pg/ml and calculated values below this were reassigned ‘zero’. Of note, 14 pg/ml IFN-gamma is equivalent to 0.35 IU/ml (www.cellestis.com). A positive response for ELISA was defined as any response >14 pg/ml for peptide pool and >100 pg/ml for the recombinant stimulation. Antigen specific responses were corrected for the background response by subtraction.

### Cell Culture

Peripheral blood mononuclear cells (PBMC) were separated over Ficoll as previously described [37]. Cells were frozen and stored in liquid nitrogen until analyzed, when they were set up at 250000/200 µl RPMI/10% FCS (R10) per well in 96U plates in the presence of various stimuli. Incubation periods ranged from overnight to 3, 5, and 7 days at 37°C in 5% CO_2_. At the end of the incubation period, plates were centrifuged to pellet the cells, cell free supernatants were collected and stored at −20°C until the ELISA was performed in batches.

### IFN-gamma ELISPOT Assay

The IFN-gamma ELISpot assay was performed as previously described [37], using mAb 1-D1K Pre-coated One-step 96-well plates (Mabtech; 3420-2ATP-10) washed with sterile PBS and blocked with R10 for 30 min at room temperature. PBMC at 2.5×10^5^ were added in 100 µl of R10 per well, followed by the respective antigenic stimuli. Control stimuli included anti-CD3 mAb at 100 ng/ml final concentration and unstimulated wells. After incubation for 16–18 h at 37°C with 5% CO_2_, plates were washed with PBS, and 100 µl of secondary antibody, mAb 7-B6-1-ALP conjugate, at 0.5 µg/ml final concentration was added. After 2 h incubation at room temperature, 100 µl substrate solution (BCIP/NBT-plus) was added until spots emerged, when the wells were washed with tap water and allowed to dry. Spot forming cells (SFC) were enumerated using the immunospot counter (CTL, Cellular Technology Ltd) and confirmed by microscope (X4). Results are quoted as spot forming cells (SFC) per 10^6^ PBMC. All ELISpot responses presented are corrected for the background.

### Antigens

Tuberculin Purified Protein derivative (PPD) was obtained from Evans Vaccines (Liverpool, UK). 10,000 U/ml stocks in PBS were stored at −80°C and used at 1000 U/ml final concentration. Recombinant rESAT-6 and rCFP-10 were obtained from Apronex, Czech Republic. The endotoxin content of these proteins was 1723 IU (EU)/mg for ESAT-6 and 260 IU (EU)/mg for CFP-10. Recombinant proteins were reconstituted in PBS/2% BSA and were used at a final concentration of 5 µg/ml. Overlapping peptides covering ESAT-6, CFP-10 and Rv2654 were 15-mers overlapping by 10 residues obtained from Peptide Protein Research Ltd, Oxford, UK. The individual peptides were solubilized in 100 µl DMSO and diluted to 10 mg/ml using PBS/2%BSA (as stock solutions). Mixtures of 17 peptides for ESAT-6 (one peptide pool), 18 peptides for CFP-10 (one peptide pool) and 14 peptides for Rv2654 (one peptide pool) were prepared in PBS/2% BSA and the final dilution of each peptide was 3 µg/ml in the assays.

### SELDI-TOF Mass Spectrometry

NP20 ProteinChip®arrays (BioRad) were primed with 5 µl of HPLC grade water (Sigma). Five microliters of neat supernantant were applied to the array surface 3 times, allowing the sample to air dry between each application. Five microliters of recombinant ESAT-6 or CFP-10 (1 mg/ml solution) were applied twice, allowing the sample to air dry between each application. The arrays were then rinsed 3 times with HPLC water and allowed to air dry for 10 min. Saturated sinapinic acid (SPA) or α-cyano-4-hydroxycinnamic acid (CHCA) were applied twice (2×0.7 µl) to each spot on the arrays, allowing the matrix to air dry between each application.

Time-of-flight spectra were generated using a PCS-4000 mass spectrometer (BioRad). Spectra from the peptide pools were obtained at a laser energy of 2000 nJ (one warming shot of 2200 nJ), a focus mass of 1500 and the matrix attenuated to 1000. Spectra from the recombinant proteins were obtained at a laser energy of 2500 nJ (one warming shot of 2750 nJ), a focus mass of 12,000 and the matrix attenuated to 5000. Ten shots were obtained per position. Mass accuracy was calibrated externally using All-in-One Peptide or Protein molecular mass standards (BioRad). Molecular weight shirts were predicted using PAWS software (Genomic Solutions).

### 1-D PAGE and Western Immunoblotting

1-D gels were performed using an Invitrogen XCell SureLock electrophoresis system. Twenty microliters of protein supernatant were separated on a 10% Bis-Tris gel using 2-(*N*-morpholino) ethanesulfonic acid (MES) buffer at 100 V for 70–80 min and compared against SeeBlue Plus 2 pre-stained protein standard (all Invitrogen, Carlsbad, CA). Protein gels were transferred to Hybond-C Extra nitrocellulose membranes (GE Healthcare, Pittsburgh, PA) in an XCell II blot electrophoretic cell (Life Technologies, Grand Island, NY) at 20 V for 60–90 min. Each membrane was blocked in PBS/3% BSA/0.1% Tween 20 for 1 hr at RT and probed with monoclonal anti-ESAT-6 (HYB 76-8CS), polyclonal anti-CFP-10 (K8493) antibodies (both from Statens Serum Institute, Copenhagen, Denmark), or Calgranulin A (FL-83,Santa Cruz Biotechnology) overnight at 4°C. Dot blots to confirm his-tagged recombinant ESAT-6 and CFP-10 were performed by adding 5 µl of supernatant onto Hybond-C Extra nitrocellulose membranes (Amersham, Amersham, UK) and a his-tagged DsbA control [38]. After blocking, the membrane was probed with Penta Anti-His Antibody Selector Kit (Qiagen) shaking at RT for 30 min.

### Statistical Analysis

The normality of data was assessed by the D’Agostino and Pearson omnibus test using Graphpad Prism 5.0 software (www.graphpad.com). Parametric continuous variables were assessed by student’s paired and unpaired t-tests, and non-parametric variables by Wilcoxon matched pairs, Kruskal Wallis test with Dunn’s post test correction or Mann Whitney U tests. Contingency analysis was by Fisher’s exact test of probability. The kappa statistic was used to test the agreement between two tests. Correlation was assessed by non-parametric Spearman correlation coefficient. Differences were considered significant when p<0.05.

## Results

### The T Cell Response to Peptide Pools is Significantly Lower than the Recombinant Proteins in the 7 Day Whole Blood Assay

**Figure 1:**
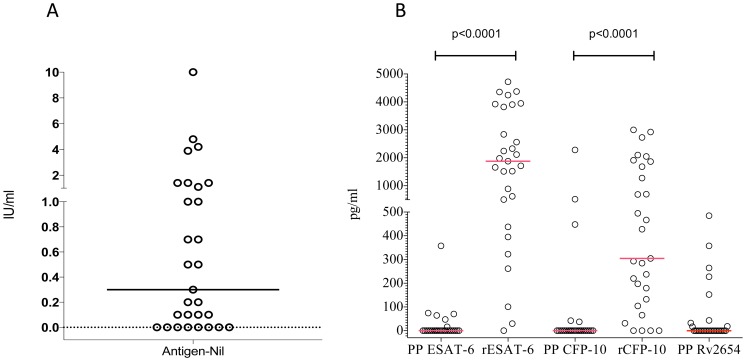
QFT-IT results, representing the Antigen-Nil IU/ml INF-gamma values (n = 29, Panel A). The solid line represents the median. **IFN-gamma response to peptide pools (covering ESAT-6, CFP-10, and Rv2654) and recombinant ESAT-6 and CFP-10 in the 7 day WBA (n = 29, Panel B).** “PP” indicates peptide pool and “r” indicates the recombinant proteins. Recombinant proteins induced significantly higher responses than the peptide pools (p<0.0001 for both ESAT-6 and CFP-10). IFN-gamma concentrations are shown as pg/ml. Lines indicate median response.

We first compared the commercially available overnight QuantiFERON TB Gold in Tube (QFT-GIT) with the widely used 7 day diluted WBA. Of the 29 tested volunteers, 14 (48%) were found positive by the QFT-GIT test (median IFN-gamma 0.3 IU/ml, IQR 0–1.25, Figure 1, Panel A). As the QFT-GIT assay incorporates TB7.7, which is Rv2654 of *Mycobacterium tuberculosis*, we included Rv2654c as a peptide pool in the initial 7-day diluted WBA. Using the 7 day diluted WBA with peptide pools as stimulants, 10/29 donors (34%) responded to at least one peptide pool. However, when recombinant proteins were used as stimulants, 27/29 (93%) donors responded to either recombinant ESAT-6 or CFP-10, and this difference was significant when compared to the peptides (p<0.0001 Wilcoxon matched pairs test, Figure 1, Panel B). The median IFN-gamma response to all ESAT-6, CFP-10 and Rv2654 peptide pools was 0 pg/ml, while the median response to the recombinant ESAT-6 and CFP-10 was 1878 (IQR 471–3863) and 305 pg/ml (IQR 118–1774) respectively (p<0.0001 for both comparisons). A significant correlation was observed between the QFT-GIT (IU/ml) and the 7 day WBA (pg/ml) response to the ESAT-6 peptide pool (r = 0.45; p = 0.017) and the recombinant CFP-10 (r = 0.59; p = 0.001) (Table 1).

**Table 1:** Correlation between QFT-GIT and IFN-gamma response in the 7-day whole blood assay.

Parameter	ESAT-6 peptide pool	CFP-10 peptide pool	Rv2654 peptide pool	rESAT-6	rCFP-10
n*	28	28	28	28	28
Spearman r	0.45	0.15	0.29	0.33	0.59
95% Confidence interval	0.08 to 0.71	−0.25 to 0.50	−0.10 to 0.60	−0.06 to 0.63	0.26 to 0.79
p value (two-tailed)	**0.017**	0.45	0.13	0.086	**0.001**

* 1 person had QFT-GIT IFN-gamma response >10 IU/ml and was therefore excluded from this analysis.

**Figure 2:**
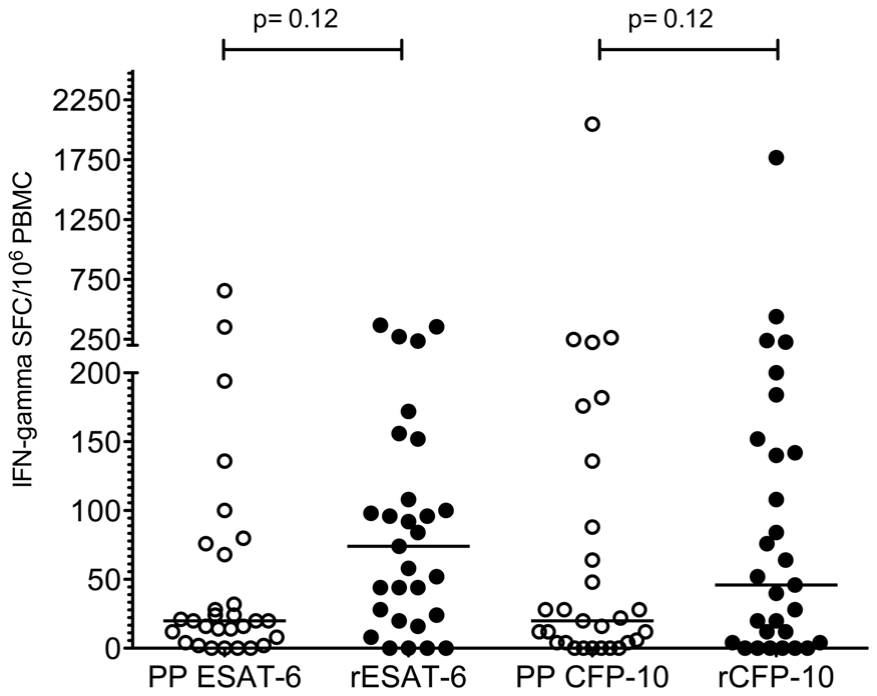
IFN-gamma response of PBMC to peptide pools and recombinant proteins in the overnight ELISpot assay (n = 29). “PP” indicates the peptide pools and “r” indicates recombinant proteins of ESAT-6 and CFP-10. No significant difference was found between the peptide pool and recombinant protein for either ESAT-6 or CFP-10 (Wilcoxon matched pairs test). Lines indicate median response.

**Figure 3:**
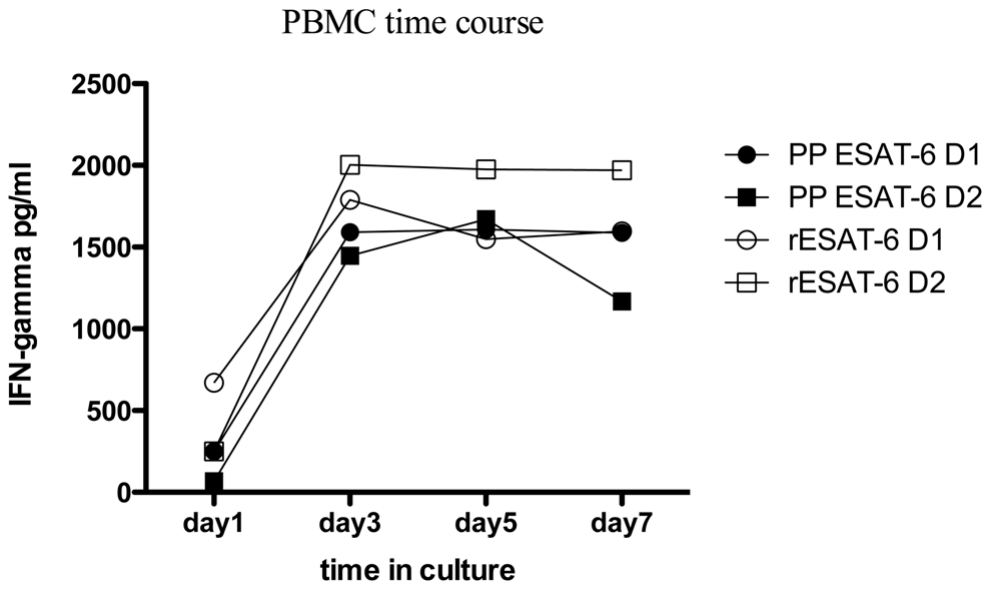
Peptides and recombinant protein have similar activity in prolonged PBMC culture. PBMC of two donors were set up with either peptide pool or recombinant ESAT-6 and cultured for 1,3,5,7 days. IFN-gamma was measured in the culture supernatant by ELISA. Filled symbols represent peptide stimulation, open symbols represent stimulation with recombinant protein. Donor 1 is represented by circles, and Donor 2 by squares.

We next compared the response between peptide pools and recombinant proteins in the overnight ELISpot assay using PBMC. As we had no recombinant Rv2654 available, these experiments were carried out using ESAT-6 and CFP-10 (peptides and recombinants) only. No difference in IFN-gamma induction was observed between peptide pools and recombinant proteins in the overnight ELISpot assay using PBMC from the same donors (Figure 2). Considering the low endotoxin content of these recombinant proteins, and the clear negative responses observed in some donors in the ELISpot assay, the possibility of contamination in the recombinant protein to induce higher IFN-gamma responses was ruled out. Additionally, experiments using PBMC from two donors stimulated with either peptide pools or the recombinant proteins for 1, 3, 5 or 7 days, showed similar concentrations of IFN-gamma secretion (Figure 3), indicating that peptides and recombinants have similar activity in prolonged PBMC based cultures.

### SELDI-TOF MS Analysis of Whole Blood Assay Supernatants

**Figure 4:**
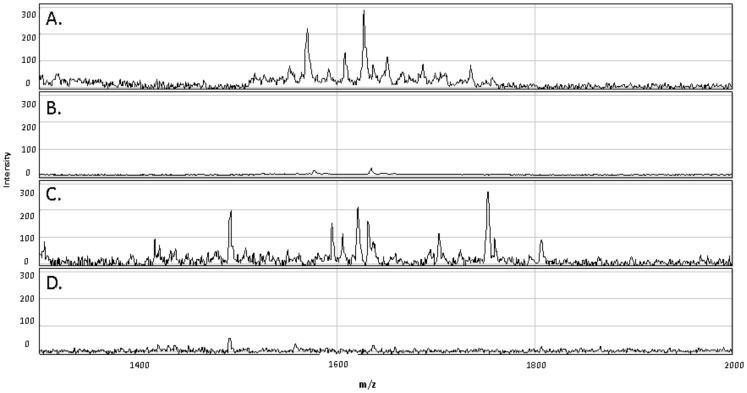
SELDI-TOF mass spectrometry analysis of whole blood supernatant stimulated with ESAT-6 and CFP-10 peptide pools. (A) ESAT-6 peptide pool stimulated whole blood at Day 0 with expected masses from 1419 Da to 1738 Da. (B) ESAT-6 peptide pool stimulated whole blood harvested after 7 days of incubation. (C) CFP-10 peptide pool stimulated whole blood at Day 0 with expected masses from 1415 Da to 1804 Da. (D) CFP-10 peptide pool stimulated whole blood harvested after 7 days of incubation.

**Figure 5:**
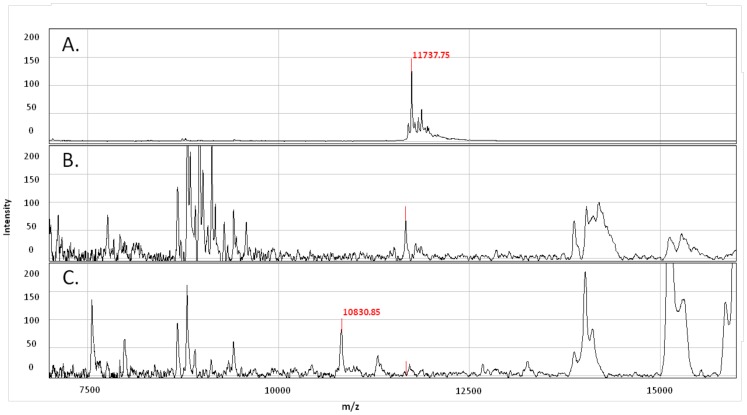
Recombinant ESAT-6 protein profiles on SELDI-TOF mass spectrometry. (A) Recombinant rESAT-6 protein. (B) Recombinant ESAT-6 stimulated whole blood at Day 0 showing a peak at the predicted molecular weight of 11.7 kDa. (C) Recombinant ESAT-6 stimulated whole blood after 7 days of incubation showing that rESAT-6 cannot be detected, while a new peak appears at 10.8 kDa.

**Figure 6:**
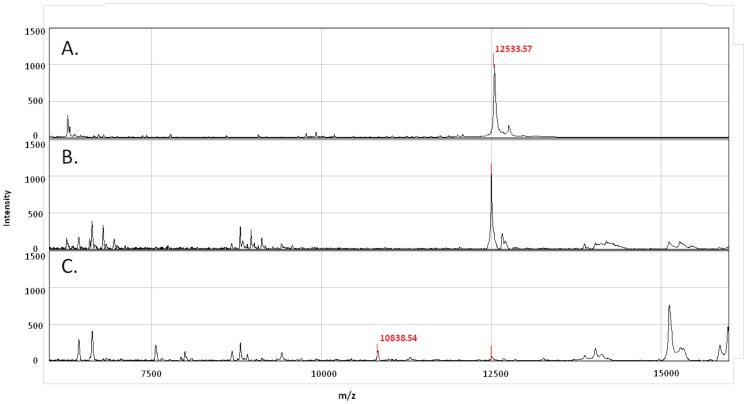
Recombinant CFP-10 protein profiles on SELDI-TOF mass spectrometry. (A) Recombinant rCFP-10 protein; (B) recombinant CFP-10 stimulated whole blood at Day 0 showing a peak at the predicted molecular weight of 12.5 kDa; (C) recombinant CFP-10 stimulated whole blood after 7 days of incubation showing that rCFP-10 cannot be detected, while a new peak appears at 10.8 kDa.

**Figure 7:**
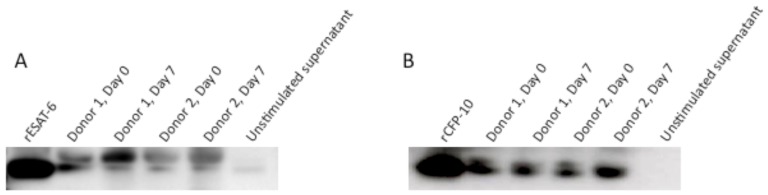
Detection of recombinant proteins by Western blot: Supernatant of whole blood stimulated with (A) rESAT-6 and (B) rCFP-10 at Day 0 and after 7 days of incubation, in 2 donors, showing the presence of both recombinant proteins after 7 days of incubation, including positive (recombinant proteins alone) and negative (supernatant of unstimulated whole blood) controls.

Because peptides are degraded more easily by proteases present in whole blood than recombinant proteins [39], [40], we hypothesized that insufficient peptide concentrations were present after 7 days of culture to continue stimulating T cells. In order to detect possible peptide degradation, we compared the SELDI-TOF mass spectrometry profiles of ESAT-6 and CFP-10 peptide pools and recombinant proteins in the supernatants of whole blood assays from 2 donors collected at Day 0 and after 7 days of incubation. As shown in Figure 4, the ESAT-6 and CFP-10 peptide peaks were present at Day 0 but not detectable after 7 days of culture, suggesting that the peptide pools were degraded during the course of the experiment. Protein profiling of recombinant ESAT-6 and CFP-10 showed a clear peak for each antigen, corresponding to the predicted molecular weight at Day 0 that was still detectable at Day 7, although present in lower concentrations (Figures 5 and 6). Due to the low levels of rESAT-6 and rCFP-10 that were detectable by mass spectroscopy after 7 days of culture, we confirmed the presence of both recombinant proteins by western blot. The results shown in Figure 7 confirm, that both recombinant proteins are still present after 7 days of incubation.

**Figure 8:**
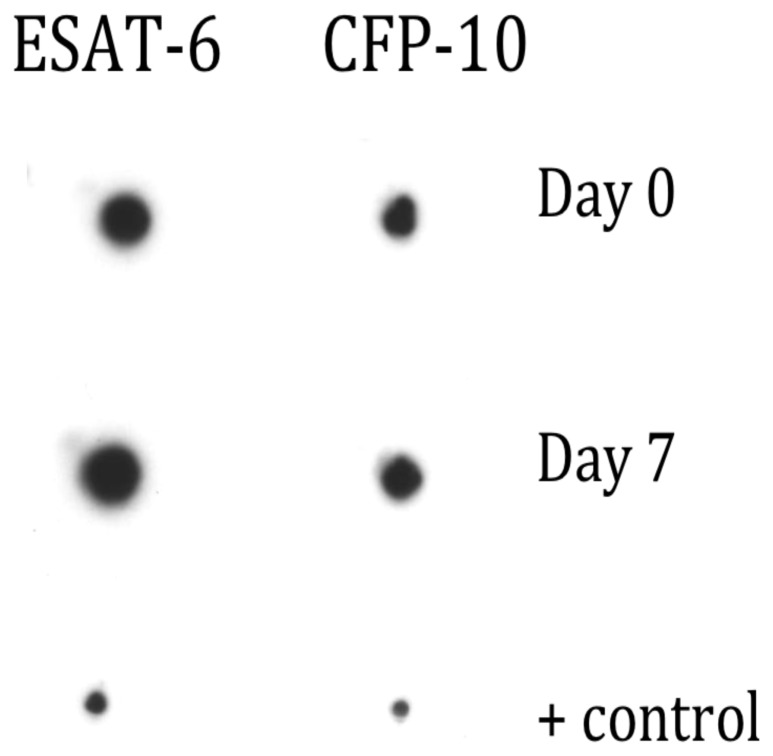
Detection of His-Tagged recombinant proteins by Western blot: in the supernatant of whole blood stimulated with rESAT-6 and rCFP-10 at Day 0 and after 7 days of incubation compared to a positive control, showing that the His-tag is detectable at both Day 0 and Day 7 of culture.

We also observed new peaks of lower molecular weight in both the ESAT-6 and CFP-10 protein profiles, after 7 days of culture, in particular a highly expressed protein at 10.8 kDa. To determine whether protein cleavage of the 3′ C-terminal His-tag would account for the new peaks of lower molecular weight, Day 0 and Day 7 supernatants from recombinant ESAT-6 and CFP-10 stimulated whole blood were separated on a 1-D PAGE gel and probed with penta-anti-His antibody (Figure 8). These results showed that the His-tag was detectable at Day 0 and after 7 days of culture for both recombinant proteins, refuting that exopeptidase degradation of the C-terminus could account for the change in mass.

**Figure 9:**
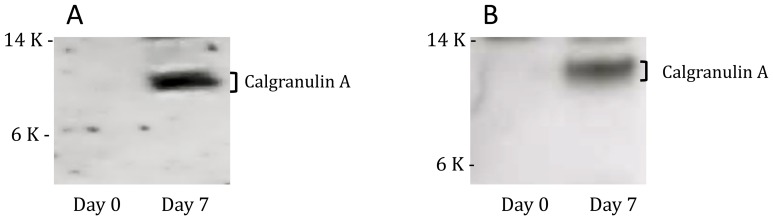
Detection of Calgranulin A (S100A8) by Western blot: Supernatant of whole blood stimulated with (A) rESAT-6 and (B) rCFP-10, was probed with Calgranulin A polycolonal antibody at Day 0 and after 7 days of incubation. The data indicates the presence of Calgranulin A after 7 days of culture, but not on Day 0.

The peak observed at 10.8 kDa had a high intensity and appeared after stimulation with either rESAT-6 or rCFP-10 proteins but not with the peptide pools. A database search for proteins previously identified at an m/z of 10.8 kDa identified Calgranulin A (S100A8) as the potential protein. Calgranulin A is an S-100 calcium-binding protein that is expressed in a variety of cell types including macrophages and neutrophils during acute inflammation. In order to determine if there was an increase in Calgranulin A after long-term culture, rESAT-6 and rCFP-10 stimulated 7-day WBA supernatants were analysed by western blot. As shown in Figure 9, whole blood stimulated with either rESAT-6 or rCFP-10 contained Calgranulin A after 7 days of culture, but not at Day 0.

## Discussion

In this study we investigated the secretion of IFN-γ in the commercially available QuantiFERON TB Gold in Tube (QFT-GIT) and long-term (7 day) whole blood assays, as well as the ELISpot assay in response to ESAT-6 and CFP-10. The performance of these two antigens was also compared in the form of synthetic overlapping peptide pools and recombinant proteins in 7 day WBA and PBMC based assays (both long term culture and ELISpot assay). Our data indicate a reduced response to peptide pools compared to recombinant proteins in the 7-day WBA, which has not been reported previously. The activity of peptide pools and recombinant proteins did not differ in PBMC cultures.

The IFN-gamma response in a 7-day culture results from proliferating cells. Therefore assays based on prolonged culture would depend on the presence of adequate amounts of antigenic stimuli (either peptide or recombinant protein) during the course of the culture period. Peptides have greater sensitivity than recombinant proteins to degradation by proteases present in cultured whole blood [39], [40]. Our SELDI-TOF mass spectrometry data suggest that peptide degradation is most probably responsible for the reduced response observed, as the peptides are degraded by day 7 and not available to stimulate the proliferating T cells.

Our data also suggest that recombinant proteins are less abundant after 7 days of whole blood culture. However, we were clearly able to detect both recombinant proteins after 7 days of culture by Western blot. We did investigate the possibility that new peaks of lower molecular weight that have appeared after 7 days, were due to C-terminal degradation of the recombinant proteins. As we were able to detect that the His-tag was present in both Day 0 and Day 7 samples, we conclude that reduced abundance of the recombinant proteins is most likely due to uptake, processing and presentation by the antigen presenting cells during the culture period.

Interestingly, one of the lower molecular weight peaks that appeared after 7 days of incubation was observed at 10.8 kDa. A database search identified Calgranulin A (S100A8) as the potential candidate, and we confirmed that this molecule is indeed detectable after 7 days of stimulation with rESAT-6 and rCFP-10. Calgranulins are endogenously expressed at various levels in myeloid cells (neutrophils, macrophages, dendritic cells, monocytes), secreted upon cellular activation and involved in a number of cellular functions, including cell growth and differentiation [41]. The secreted form by neutrophils enhances CD11b expression on human monocytes, and is a potent chemotactic for leukocytes [42]. S100A8 detected by SELDI at day 7 could be the result of the maturation process that adherent whole blood derived monocytes go through during the incubation period, maturing into monocyte derived macrophages, and acting as antigen presenting cells. While this was not the focus of the present manuscript, the significance of calgranulin A needs to be explored in further studies.

Our results of recombinant proteins used in 7 day WBA as stimulants are in line with other published studies using the same assay. The majority of published studies use 7 day WBA with recombinant proteins and 24 hrs diluted/undiluted WBA with peptide pools. Two main recent antigen discovery papers from the Ottenhoff group use recombinant proteins [21], [43]. A study looking at diagnosing leprosy with synthetic peptides [44] uses synthetic 20-mer peptides overlapping by 10 amino acids in PBMC cultured for 6 days, which is the assay system where we showed no difference between peptide pools and recombinant proteins. We are aware of one exception [45], which evaluates and describes novel MTB infection phase-dependent antigens in TB patients and household contacts. The authors included 8 peptide pools along with other recombinant proteins in a 7 day diluted whole blood assay similar to our assay. They describe the recognition of the 8 peptide pools as “poor” in both TB patients and household contacts.

While our data is preliminary with a number of limitations, and further confirmatory studies are warranted, we conclude that assessment of vaccine candidates in the form of peptides should be tested in short term assays, while long-term whole blood based assays should be based on recombinant protein formulations.

## References

[1] Hanekom WA, Dockrell HM, Ottenhoff TH, Doherty TM, Fletcher H, et al (2008) Immunological Outcomes of New Tuberculosis Vaccine Trials: WHO Panel Recommendations. PLoS Med 5: e145. 18597551 10.1371/journal.pmed.0050145 PMC2443198

[2] Pai M, Zwerling A, Menzies D (2008) Systematic review: T-cell-based assays for the diagnosis of latent tuberculosis infection: an update. Ann Intern Med 149: 177–184. 18593687 10.7326/0003-4819-149-3-200808050-00241 PMC2951987

[3] Lalvani A, Pareek M (2009) A 100 year update on diagnosis of tuberculosis infection. Br Med Bull 93: 69–84. 19926636 10.1093/bmb/ldp039

[4] Ravn P, Demissie A, Eguale T, Wondwosson H, Lein D, et al (1999) Human T cell responses to the ESAT-6 antigen from *Mycobacterium tuberculosis* . J Infect Dis 179: 637–645. 9952370 10.1086/314640

[5] Arend SM, Geluk A, van Meijgaarden KE, van Dissel JT, Theisen M, et al (2000) Antigenic equivalence of human T-cell responses to *Mycobacterium tuberculosis*-specific RD1-encoded protein antigens ESAT-6 and culture filtrate protein 10 and to mixtures of synthetic peptides. Infect Immun 68: 3314–3321. 10816479 10.1128/iai.68.6.3314-3321.2000 PMC97589

[6] Tena-Coki NG, Scriba TJ, Peteni N, Eley B, Wilkinson RJ, et al (2010) CD4 and CD8 T Cell Responses to Mycobacterial Antigens in African Children. Am J Respir Crit Care Med. 182: 120–129. 10.1164/rccm.200912-1862OC PMC2902756 20224065

[7] Soares AP, Scriba TJ, Joseph S, Harbacheuski R, Murray RA, et al (2008) Bacillus Calmette-Guerin vaccination of human newborns induces T cells with complex cytokine and phenotypic profiles. J Immunol 180: 3569–3577. 18292584 10.4049/jimmunol.180.5.3569 PMC2842001

[8] Hanekom WA, Hughes J, Mavinkurve M, Mendillo M, Watkins M, et al (2004) Novel application of a whole blood intracellular cytokine detection assay to quantitate specific T-cell frequency in field studies. J Immunol Methods 291: 185–195. 15345316 10.1016/j.jim.2004.06.010

[9] Scholvinck E, Wilkinson KA, Whelan AO, Martineau AR, Levin M, et al (2004) Gamma interferon-based immunodiagnosis of tuberculosis: comparison between whole-blood and enzyme-linked immunospot methods. J Clin Microbiol 42: 829–831. 14766863 10.1128/JCM.42.2.829-831.2004 PMC344457

[10] Bertholet S, Ireton GC, Kahn M, Guderian J, Mohamath R, et al (2008) Identification of human T cell antigens for the development of vaccines against *Mycobacterium tuberculosis* . J Immunol 181: 7948–7957. 19017986 10.4049/jimmunol.181.11.7948 PMC2586986

[11] Wilkinson RJ, Wilkinson KA, De Smet KA, Haslov K, Pasvol G, et al (1998) Human T- and B-cell reactivity to the 16 kDa alpha-crystallin protein of *Mycobacterium tuberculosis* . Scand J Immunol 48: 403–409. 9790311 10.1046/j.1365-3083.1998.00420.x

[12] Wilkinson RJ, Vordermeier HM, Wilkinson KA, Sjolund A, Moreno C, et al (1998) Peptide-specific T cell response to *Mycobacterium tuberculosis*: clinical spectrum, compartmentalization, and effect of chemotherapy. J Infect Dis 178: 760–768. 9728545 10.1086/515336

[13] Weir RE, Morgan AR, Britton WJ, Butlin CR, Dockrell HM (1994) Development of a whole blood assay to measure T cell responses to leprosy: a new tool for immuno-epidemiological field studies of leprosy immunity. J Immunol Methods 176: 93–101. 7963598 10.1016/0022-1759(94)90353-0

[14] Todryk SM, Pathan AA, Keating S, Porter DW, Berthoud T, et al (2009) The relationship between human effector and memory T cells measured by ex vivo and cultured ELISPOT following recent and distal priming. Immunology 128: 83–91. 19689738 10.1111/j.1365-2567.2009.03073.x PMC2747141

[15] Jurcevic S, Hills A, Pasvol G, Davidson RN, Ivanyi J, et al (1996) T cell responses to a mixture of *Mycobacterium tuberculosis* peptides with complementary HLA-DR binding profiles. Clin Exp Immunol 105: 416–421. 8809128 10.1046/j.1365-2249.1996.d01-791.x PMC2200523

[16] Black GF, Weir RE, Floyd S, Bliss L, Warndorff DK, et al (2002) BCG-induced increase in interferon-gamma response to mycobacterial antigens and efficacy of BCG vaccination in Malawi and the UK: two randomised controlled studies. Lancet 359: 1393–1401. 11978337 10.1016/S0140-6736(02)08353-8

[17] Beveridge NE, Price DA, Casazza JP, Pathan AA, Sander CR, et al (2007) Immunisation with BCG and recombinant MVA85A induces long-lasting, polyfunctional *Mycobacterium tuberculosis*-specific CD4+ memory T lymphocyte populations. Eur J Immunol 37: 3089–3100. 17948267 10.1002/eji.200737504 PMC2365909

[18] Sidders B, Pirson C, Hogarth PJ, Hewinson RG, Stoker NG, et al (2008) Screening of highly expressed mycobacterial genes identifies Rv3615c as a useful differential diagnostic antigen for the *Mycobacterium tuberculosis* complex. Infect Immun 76: 3932–3939. 18519559 10.1128/IAI.00150-08 PMC2519431

[19] Ewer K, Cockle P, Gordon S, Mansoor H, Govaerts M, et al (2006) Antigen mining with iterative genome screens identifies novel diagnostics for the *Mycobacterium tuberculosis* complex. Clin Vaccine Immunol 13: 90–97. 16426005 10.1128/CVI.13.1.90-97.2006 PMC1356633

[20] Brock I, Weldingh K, Leyten EM, Arend SM, Ravn P, et al (2004) Specific T-cell epitopes for immunoassay-based diagnosis of *Mycobacterium tuberculosis* infection. J Clin Microbiol 42: 2379–2387. 15184408 10.1128/JCM.42.6.2379-2387.2004 PMC427833

[21] Black GF, Thiel BA, Ota MO, Parida SK, Adegbola R, et al (2009) Immunogenicity of novel DosR regulon-encoded candidate antigens of *Mycobacterium tuberculosis* in three high-burden populations in Africa. Clin Vaccine Immunol 16: 1203–1212. 19553548 10.1128/CVI.00111-09 PMC2725533

[22] Agger EM, Brock I, Okkels LM, Arend SM, Aagaard CS, et al (2003) Human T-cell responses to the RD1-encoded protein TB27.4 (Rv3878) from *Mycobacterium tuberculosis* . Immunology 110: 507–512. 14632649 10.1111/j.1365-2567.2003.01763.x PMC1783067

[23] Sutherland JS, de Jong BC, Jeffries DJ, Adetifa IM, Ota MO (2010) Production of TNF-alpha, IL-12(p40) and IL-17 can discriminate between active TB disease and latent infection in a West African cohort. PLoS One 5: e12365. 20811496 10.1371/journal.pone.0012365 PMC2927558

[24] Leyten EM, Lin MY, Franken KL, Friggen AH, Prins C, et al (2006) Human T-cell responses to 25 novel antigens encoded by genes of the dormancy regulon of *Mycobacterium tuberculosis* . Microbes Infect 8: 2052–2060. 16931093 10.1016/j.micinf.2006.03.018

[25] Millington KA, Innes JA, Hackforth S, Hinks TS, Deeks JJ, et al (2007) Dynamic relationship between IFN-gamma and IL-2 profile of *Mycobacterium tuberculosis*-specific T cells and antigen load. J Immunol 178: 5217–5226. 17404305 10.4049/jimmunol.178.8.5217 PMC2743164

[26] Aagaard C, Brock I, Olsen A, Ottenhoff TH, Weldingh K, et al (2004) Mapping immune reactivity toward Rv2653 and Rv2654: two novel low-molecular-mass antigens found specifically in the *Mycobacterium tuberculosis* complex. J Infect Dis 189: 812–819. 14976597 10.1086/381679

[27] Wilkinson RJ, Haslov K, Rappuoli R, Giovannoni F, Narayanan PR, et al (1997) Evaluation of the recombinant 38-kilodalton antigen of *Mycobacterium tuberculosis* as a potential immunodiagnostic reagent. J Clin Microbiol 35: 553–557. 9041387 10.1128/jcm.35.3.553-557.1997 PMC229625

[28] Schuck SD, Mueller H, Kunitz F, Neher A, Hoffmann H, et al (2009) Identification of T-cell antigens specific for latent *Mycobacterium tuberculosis* infection. PLoS One 4: e5590. 19440342 10.1371/journal.pone.0005590 PMC2680040

[29] Lin MY, Reddy TB, Arend SM, Friggen AH, Franken KL, et al (2009) Cross-reactive immunity to *Mycobacterium tuberculosis* DosR regulon-encoded antigens in individuals infected with environmental, nontuberculous mycobacteria. Infect Immun 77: 5071–5079. 19737909 10.1128/IAI.00457-09 PMC2772522

[30] Chen J, Su X, Zhang Y, Wang S, Shao L, et al (2009) Novel recombinant RD2- and RD11-encoded *Mycobacterium tuberculosis* antigens are potential candidates for diagnosis of tuberculosis infections in BCG-vaccinated individuals. Microbes Infect 11: 876–885. 19467342 10.1016/j.micinf.2009.05.008

[31] Sallusto F, Lenig D, Forster R, Lipp M, Lanzavecchia A (1999) Two subsets of memory T lymphocytes with distinct homing potentials and effector functions. Nature 401: 708–712. 10537110 10.1038/44385

[32] Godkin AJ, Thomas HC, Openshaw PJ (2002) Evolution of epitope-specific memory CD4(+) T cells after clearance of hepatitis C virus. J Immunol 169: 2210–2214. 12165552 10.4049/jimmunol.169.4.2210

[33] Leyten EM, Arend SM, Prins C, Cobelens FG, Ottenhoff TH, et al (2007) Discrepancy between *Mycobacterium tuberculosis*-specific gamma interferon release assays using short and prolonged in vitro incubation. Clin Vaccine Immunol 14: 880–885. 17507543 10.1128/CVI.00132-07 PMC1951056

[34] Beveridge NE, Fletcher HA, Hughes J, Pathan AA, Scriba TJ, et al (2008) A comparison of IFNgamma detection methods used in tuberculosis vaccine trials. Tuberculosis (Edinb) 88: 631–640. 18801705 10.1016/j.tube.2008.06.005

[35] Rangaka MX, Diwakar L, Seldon R, van Cutsem G, Meintjes GA, et al (2007) Clinical, immunological, and epidemiological importance of antituberculosis T cell responses in HIV-infected Africans. Clin Infect Dis 44: 1639–1646. 17516410 10.1086/518234

[36] Connell TG, Shey MS, Seldon R, Rangaka MX, van Cutsem G, et al (2007) Enhanced ex vivo stimulation of *Mycobacterium tuberculosis*-specific T cells in human immunodeficiency virus-infected persons via antigen delivery by the Bordetella pertussis adenylate cyclase vector. Clin Vaccine Immunol 14: 847–854. 17522328 10.1128/CVI.00041-07 PMC1951068

[37] Gideon HP, Wilkinson KA, Rustad TR, Oni T, Guio H, et al (2010) Hypoxia induces an immunodominant target of tuberculosis specific T cells absent from common BCG vaccines. PLoS Pathog 6: e1001237. 21203487 10.1371/journal.ppat.1001237 PMC3009603

[38] Sinha S, Ambur OH, Langford PR, Tonjum T, Kroll JS (2008) Reduced DNA binding and uptake in the absence of DsbA1 and DsbA2 of Neisseria meningitidis due to inefficient folding of the outer-membrane secretin PilQ. Microbiology 154: 217–225. 18174140 10.1099/mic.0.2007/010496-0

[39] Yeaman MR, Gank KD, Bayer AS, Brass EP (2002) Synthetic peptides that exert antimicrobial activities in whole blood and blood-derived matrices. Antimicrob Agents Chemother 46: 3883–3891. 12435692 10.1128/AAC.46.12.3883-3891.2002 PMC132762

[40] Nusslein K, Arnt L, Rennie J, Owens C, Tew GN (2006) Broad-spectrum antibacterial activity by a novel abiogenic peptide mimic. Microbiology 152: 1913–1918. 16804167 10.1099/mic.0.28812-0

[41] Hofmann Bowman MA, Schmidt AM (2011) S100/calgranulins EN-RAGEing the blood vessels: implications for inflammatory responses and atherosclerosis. Am J Cardiovasc Dis 1: 92–100. 22200033 PMC3244046

[42] Donato R (2003) Intracellular and extracellular roles of S100 proteins. Microsc Res Tech 60: 540–551. 12645002 10.1002/jemt.10296

[43] Commandeur S, van Meijgaarden KE, Prins C, Pichugin AV, Dijkman K, et al (2013) An unbiased genome-wide *Mycobacterium tuberculosis* gene expression approach to discover antigens targeted by human T cells expressed during pulmonary infection. J Immunol 190: 1659–1671. 23319735 10.4049/jimmunol.1201593

[44] Geluk A, van der Ploeg J, Teles RO, Franken KL, Prins C, et al (2008) Rational combination of peptides derived from different *Mycobacterium leprae* proteins improves sensitivity for immunodiagnosis of *M. leprae* infection. Clin Vaccine Immunol 15: 522–533. 18199740 10.1128/CVI.00432-07 PMC2268264

[45] Chegou NN, Black GF, Loxton AG, Stanley K, Essone PN, et al (2012) Potential of novel *Mycobacterium tuberculosis* infection phase-dependent antigens in the diagnosis of TB disease in a high burden setting. BMC Infect Dis 12: 10. 22260319 10.1186/1471-2334-12-10 PMC3282638

