# Correction: Impairment of IFN-Gamma Response to Synthetic Peptides of *Mycobacterium tuberculosis* in a 7-Day Whole Blood Assay

**DOI:** 10.1371/annotation/c91135d7-8af0-495f-b33c-391991a91aff

**Published:** 2013-10-04

**Authors:** Hannah Priyadarshini Gideon, Melissa Shea Hamilton, Kathryn Wood, Dominique Pepper, Tolu Oni, Ronnett Seldon, Claire Banwell, Paul R. Langford, Robert J. Wilkinson, Katalin A. Wilkinson

There was an error in the affiliation of the seventh author, Claire Banwell. The correct affiliation for this author is: Brighton and Sussex Medical School, University of Sussex, Brighton, United Kingdom 

